# Supplementary material for: Downsizing and purchases of psychotropic drugs: A longitudinal study of stayers, changers and unemployed
Source: PLoS One. 2023 Dec 8;18(12):e0295383. doi: 10.1371/journal.pone.0295383 (PMC10707493; doi:10.1371/journal.pone.0295383)
Supplement: S1 Appendix — (DOCX) [file pone.0295383.s001.docx]

*Script for general estimating equations (GEE) estimating the association between exposure to downsizing and purchases of anxiolytics and sedatives,

among stayers, changers and unemployed (defined by year after downsizing) vs. an unexposed group;

*key variables:

exposure = down_exp2020 (0,1)

outcomes= N05b_dik (0,1) redeemed anxiolytics

N05c_dik (0,1) redeemed sedatives

covariates=

co_rel = year in relation to the exposure (-4 to +4)

year = calendar year

kon = sex (1,2)

pr_sick_hist= history of sick leave or disability pension 2 y pr to downsizing (0,1)

ageprc =age categories (1,2,3=oldest)

edupr =educational attainment (1,2,3,4=highest)

fampr = living with a partner and/or with children at home (0,1,2,3,4)

regionpr= region of living (1,2,3)

ndoh= (1,2,3,4,5) cohorts of the unexposed

* For N05b (anxiolytics) and N05c (sedatives), respectively:

*1) GEE stratified by employment status (stayer, changer, unemployed, not exposed), crude and adjusted analysis;

*2) contrast against the unexposed for determine trend differences;

*3) sensitivity analysis: stratification by prior sick leave;

***************

Stayers;

***************;

**proc** **sql**;

create table exp_stayer_new as

select *

from downs.long_2020

where emplstat_new=**1**;

**quit**;

***************

Changers;

***************;

**proc** **sql**;

create table exp_changer_new as

select *

from downs.long_2020

where emplstat_new=**2**;

**quit**;

***************

Unemployed;

***************;

**proc** **sql**;

create table exp_unemployed_new as

select *

from downs.long_2020

where emplstat_new=**0**;

**quit**;

***************

Not exposed;

***************;

**proc** **sql**;

create table not_exp as

select *

from downs.fullsample_9periods

where ndcoh ne **.**;

**quit**;

*N05B (anxiolytics);

*N05b crude;

*repeat same analysis but switch data set into the other empl groups+ the unexposed, and outcome n05c_dik;

**proc** **genmod** data= exp_stayer_new descend; *REPEAT for changers, unemployed and unexposed;

class Lopnr co_rel year;

model N05b_dik = co_rel year / dist=bin link=logit; *repeat for N05c_dik, sedatives;

lsmeans co_rel / diff cl;

estimate '-3 vs -4' co_rel -**1** **1** **0** **0** **0** **0** **0** **0** **0** / exp;

estimate '-1 vs -3' co_rel **0** -**1** **0** **1** **0** **0** **0** **0** **0** / exp;

estimate '1 vs -1' co_rel **0** **0** **0** -**1** **0** **1** **0** **0** **0** / exp;

estimate '4 vs 1' co_rel **0** **0** **0** **0** **0** -**1** **0** **0** **1** / exp;

repeated subject=Lopnr / within=co_rel type=ar;

ods output lsmeans=tempout;

**run**;

**data** tempout2;

set tempout;

estprev=**100***exp(Estimate);

low=**100***exp(lower);

upp=**100***exp(upper);

**proc** **print** data=tempout2;

var co_rel estprev low upp;

**run**;

*N05b adj;

**proc** **genmod** data= exp_stayer_new descend; *REPLACE data sets as above ;

class Lopnr co_rel year kon ageprc edupr pr_sick_hist fampr regionpr ;

model N05b_dik = co_rel year kon ageprc edupr pr_sick_hist fampr regionpr/ dist=bin link=logit;*replace with N05c_dik for sedatives;

lsmeans co_rel / diff cl;

estimate '-3 vs -4' co_rel -**1** **1** **0** **0** **0** **0** **0** **0** **0** / exp;

estimate '-1 vs -3' co_rel **0** -**1** **0** **1** **0** **0** **0** **0** **0** / exp;

estimate '1 vs -1' co_rel **0** **0** **0** -**1** **0** **1** **0** **0** **0** / exp;

estimate '4 vs 1' co_rel **0** **0** **0** **0** **0** -**1** **0** **0** **1** / exp;

repeated subject=Lopnr / within=co_rel type=ar;

ods output lsmeans=tempout;

**run**;

**data** tempout2;

set tempout;

estprev=**100***exp(Estimate);

low=**100***exp(lower);

upp=**100***exp(upper);

**proc** **print** data=tempout2;

var co_rel estprev low upp;

**run**;

***Test of contrast between each of the exposed groups against the not exposed group;

*joint data set and single variable of exposure;

**data** downs.long_2020;

set downs.long_2020;

if ndcoh ne **.** then down_exp2020=**0**; /*not exposed*/

else if emplstat_new=**1** then down_exp2020=**1**; /*stayer*/

else if emplstat_new=**2** then down_exp2020=**2**; /*changer*/

else if emplstat_new=**0** then down_exp2020=**3**; /*unemployed*/

**run**;

*Here N05b_dik (anxiolytics) as outcome, replace with N05C_dik as outcome for sedatives;

*n05b, adj;

**proc** **genmod** data= downs.long_2020 descend;

class Lopnr down_exp2020 co_rel year kon pr_sick_hist ageprc edupr fampr regionpr;

model N05b_dik = down_exp2020 co_rel year kon pr_sick_hist ageprc edupr fampr regionpr down_exp2020*co_rel down_exp2020*co_rel*pr_sick_hist/ dist=bin link=logit wald;

*year -4 vs -3;

estimate 'not exp vs stayer -4 vs -3' down_exp2020*co_rel **1** -**1** **0** **0** **0** **0** **0** **0** **0**

-**1** **1** **0** **0** **0** **0** **0** **0** **0**

**0** **0** **0** **0** **0** **0** **0** **0** **0**

**0** **0** **0** **0** **0** **0** **0** **0** **0** /exp e; /*not exposed vs stayers year -4 against year -3*/

estimate 'not exp vs changer -4 vs -3' down_exp2020*co_rel **1** -**1** **0** **0** **0** **0** **0** **0** **0**

**0** **0** **0** **0** **0** **0** **0** **0** **0**

-**1** **1** **0** **0** **0** **0** **0** **0** **0**

**0** **0** **0** **0** **0** **0** **0** **0** **0** /exp e; /*not exposed vs changer year -4 against year -3*/

estimate 'not exp vs unemp -4 vs -3' down_exp2020*co_rel **1** -**1** **0** **0** **0** **0** **0** **0** **0**

**0** **0** **0** **0** **0** **0** **0** **0** **0**

**0** **0** **0** **0** **0** **0** **0** **0** **0**

-**1** **1** **0** **0** **0** **0** **0** **0** **0** /exp e; /*not exposed vs unemployed year -4 against year -3*/

repeated subject=Lopnr / within=co_rel type=ar;

ods output lsmeans=tempout;

**run**;

**proc** **genmod** data= downs.long_2020 descend;

class Lopnr down_exp2020 co_rel year kon pr_sick_hist ageprc edupr fampr regionpr;

model N05b_dik = down_exp2020 co_rel year kon pr_sick_hist ageprc edupr fampr regionpr down_exp2020*co_rel/ dist=bin link=logit wald;

*year -3 vs -1;

estimate 'not exp vs stayer -3 vs -1' down_exp2020*co_rel **0** **1** **0** -**1** **0** **0** **0** **0** **0**

**0** -**1** **0** **1** **0** **0** **0** **0** **0**

**0** **0** **0** **0** **0** **0** **0** **0** **0**

**0** **0** **0** **0** **0** **0** **0** **0** **0** /exp e; /*not exposed vs stayers year -3 against year -1*/

estimate 'not exp vs changer -3 vs -1' down_exp2020*co_rel **0** **1** **0** -**1** **0** **0** **0** **0** **0**

**0** **0** **0** **0** **0** **0** **0** **0** **0**

**0** -**1** **0** **1** **0** **0** **0** **0** **0**

**0** **0** **0** **0** **0** **0** **0** **0** **0** /exp e; /*not exposed vs changer year -3 against year -1*/

estimate 'not exp vs unemp -3 vs 1' down_exp2020*co_rel **0** **1** **0** -**1** **0** **0** **0** **0** **0**

**0** **0** **0** **0** **0** **0** **0** **0** **0**

**0** **0** **0** **0** **0** **0** **0** **0** **0**

**0** -**1** **0** **1** **0** **0** **0** **0** **0** /exp e; /*not exposed vs unemployed year -1 against year 1*/

repeated subject=Lopnr / within=co_rel type=ar;

ods output lsmeans=tempout;

**run**;

**proc** **genmod** data= downs.long_2020 descend;

class Lopnr down_exp2020 co_rel year kon pr_sick_hist ageprc edupr fampr regionpr;

model N05b_dik = down_exp2020 co_rel year kon pr_sick_hist ageprc edupr fampr regionpr down_exp2020*co_rel / dist=bin link=logit wald;

*year -1 vs 1;

estimate 'not exp vs stayer -1 vs 1' down_exp2020*co_rel **0** **0** **0** **1** **0** -**1** **0** **0** **0**

**0** **0** **0** -**1** **0** **1** **0** **0** **0**

**0** **0** **0** **0** **0** **0** **0** **0** **0**

**0** **0** **0** **0** **0** **0** **0** **0** **0** /exp e; /*not exposed vs stayers year -1 against year 1*/

estimate 'not exp vs changer -1 vs 1' down_exp2020*co_rel **0** **0** **0** **1** **0** -**1** **0** **0** **0**

**0** **0** **0** **0** **0** **0** **0** **0** **0**

**0** **0** **0** -**1** **0** **1** **0** **0** **0**

**0** **0** **0** **0** **0** **0** **0** **0** **0** /exp e; /*not exposed vs changer year -1 against year 1*/

estimate 'not exp vs unemp -1 vs 1' down_exp2020*co_rel **0** **0** **0** **1** **0** -**1** **0** **0** **0**

**0** **0** **0** **0** **0** **0** **0** **0** **0**

**0** **0** **0** **0** **0** **0** **0** **0** **0**

**0** **0** **0** -**1** **0** **1** **0** **0** **0** /exp e; /*not exposed vs unemployed year -1 against year 1*/

repeated subject=Lopnr / within=co_rel type=ar;

ods output lsmeans=tempout;

**run**;

**proc** **genmod** data= downs.long_2020 descend;

class Lopnr down_exp2020 co_rel year kon pr_sick_hist ageprc edupr fampr regionpr;

model N05b_dik = down_exp2020 co_rel year kon pr_sick_hist ageprc edupr fampr regionpr down_exp2020*co_rel / dist=bin link=logit wald;

*year 1 vs 4;

estimate 'not exp vs stayer 1 vs 4' down_exp2020*co_rel **0** **0** **0** **0** **0** **1** **0** **0** -**1**

**0** **0** **0** **0** **0** -**1** **0** **0** **1**

**0** **0** **0** **0** **0** **0** **0** **0** **0**

**0** **0** **0** **0** **0** **0** **0** **0** **0** /exp e; /*not exposed vs stayers year 1 against year 4*/

estimate 'not exp vs changer 1 vs 4' down_exp2020*co_rel **0** **0** **0** **0** **0** **1** **0** **0** -**1**

**0** **0** **0** **0** **0** **0** **0** **0** **0**

**0** **0** **0** **0** **0** -**1** **0** **0** **1**

**0** **0** **0** **0** **0** **0** **0** **0** **0** /exp e; /*not exposed vs changer year 1 against year 4*/

estimate 'not exp vs unemp 1 vs 4' down_exp2020*co_rel **0** **0** **0** **0** **0** **1** **0** **0** -**1**

**0** **0** **0** **0** **0** **0** **0** **0** **0**

**0** **0** **0** **0** **0** **0** **0** **0** **0**

**0** **0** **0** **0** **0** -**1** **0** **0** **1** /exp e; /*not exposed vs unemployed year 1 against year 4*/

repeated subject=Lopnr / within=co_rel type=ar;

ods output lsmeans=tempout;

**run**;

*********************************

Sensitivity analysis by prior sickness absence history;

**********************************;

* stayers +

prior sick leave;

**proc** **sql**;

create table exp_stayer_sick as

select *

from downs.long_2020

where emplstat_new=**1** and pr_sick_hist=**1**;

**quit**;

*

*NO previous sick or dp;

**proc** **sql**;

create table exp_stayer_nosick as

select *

from downs.long_2020

where emplstat_new=**1** and pr_sick_hist=**0**;

**quit**;

* changers

+ prior sick leave;

**proc** **sql**;

create table exp_changer_sick as

select *

from downs.long_2020

where emplstat_new=**2** and pr_sick_hist=**1**;

**quit**;

*

*NO previous sick or dp;

**proc** **sql**;

create table exp_changer_nosick as

select *

from downs.long_2020

where emplstat_new=**2** and pr_sick_hist=**0**;

**quit**;

* unemployed

+ prior sick leave;

**proc** **sql**;

create table exp_unemployed_sick as

select *

from downs.long_2020

where emplstat_new=**0** and pr_sick_hist=**1**;

**quit**;

*

*NO previous sick or dp;

**proc** **sql**;

create table exp_unemployed_nosick as

select *

from downs.long_2020

where emplstat_new=**0** and pr_sick_hist=**0**;

**quit**;

*Not exposed

+ prior sick;

**proc** **sql**;

create table not_exp_sick as

select *

from downs.fullsample_9periods

where ndcoh ne **.** and pr_sick_hist=**1**;

**quit**;

* no sick leave;

**proc** **sql**;

create table not_exp_nosick as

select *

from downs.fullsample_9periods

where ndcoh ne **.** and pr_sick_hist=**0**;

**quit**;

****************************************;

*N05C;

*N05c crude;

**proc** **genmod** data= exp_stayer_sick descend; *replace w data set from above- according to changer, unempl, not exp and pr_sick_hist=0 or 1;

class Lopnr co_rel year;

model N05c_dik = co_rel year / dist=bin link=logit; *replace w N05b_dik;

lsmeans co_rel / diff cl;

estimate '-3 vs -4' co_rel -**1** **1** **0** **0** **0** **0** **0** **0** **0** / exp;

estimate '-1 vs -3' co_rel **0** -**1** **0** **1** **0** **0** **0** **0** **0** / exp;

estimate '1 vs -1' co_rel **0** **0** **0** -**1** **0** **1** **0** **0** **0** / exp;

estimate '4 vs 1' co_rel **0** **0** **0** **0** **0** -**1** **0** **0** **1** / exp;

repeated subject=Lopnr / within=co_rel type=ar;

ods output lsmeans=tempout;

**run**;

**data** tempout2;

set tempout;

estprev=**100***exp(Estimate);

low=**100***exp(lower);

upp=**100***exp(upper);

**proc** **print** data=tempout2;

var co_rel estprev low upp;

**run**;

*N05c adj;

**proc** **genmod** data= exp_stayer_sick descend;

class Lopnr co_rel year kon ageprc edupr fampr regionpr ; *replace w data set from above- according to changer, unempl, not exp and pr_sick_hist=0 or 1;

model N05c_dik = co_rel year kon ageprc edupr fampr regionpr/ dist=bin link=logit; *Switch to N05b_dik;

lsmeans co_rel / diff cl;

estimate '-3 vs -4' co_rel -**1** **1** **0** **0** **0** **0** **0** **0** **0** / exp;

estimate '-1 vs -3' co_rel **0** -**1** **0** **1** **0** **0** **0** **0** **0** / exp;

estimate '1 vs -1' co_rel **0** **0** **0** -**1** **0** **1** **0** **0** **0** / exp;

estimate '4 vs 1' co_rel **0** **0** **0** **0** **0** -**1** **0** **0** **1** / exp;

repeated subject=Lopnr / within=co_rel type=ar;

ods output lsmeans=tempout;

**run**;

**data** tempout2;

set tempout;

estprev=**100***exp(Estimate);

low=**100***exp(lower);

upp=**100***exp(upper);

**proc** **print** data=tempout2;

var co_rel estprev low upp;

**run**;

***Contrast test between exposed and unexposed, stratified by prior sickness absence;

*perform same analysis but switch data set into no_previous sick and outcome n05c_dik;

**proc** **genmod** data= prev_sick descend;*change to no_previous sick;

class Lopnr down_exp2020 co_rel year kon ageprc edupr fampr regionpr;

model N05b_dik = down_exp2020 co_rel year kon ageprc edupr fampr regionpr down_exp*co_rel / dist=bin link=logit wald;

*year -4 vs -3;

estimate 'not exp vs stayer -4 vs -3' down_exp2020*co_rel **1** -**1** **0** **0** **0** **0** **0** **0** **0**

-**1** **1** **0** **0** **0** **0** **0** **0** **0**

**0** **0** **0** **0** **0** **0** **0** **0** **0**

**0** **0** **0** **0** **0** **0** **0** **0** **0** /exp e; /*not exposed vs stayers year -4 against year -3*/

estimate 'not exp vs changer -4 vs -3' down_exp2020*co_rel **1** -**1** **0** **0** **0** **0** **0** **0** **0**

**0** **0** **0** **0** **0** **0** **0** **0** **0**

-**1** **1** **0** **0** **0** **0** **0** **0** **0**

**0** **0** **0** **0** **0** **0** **0** **0** **0** /exp e; /*not exposed vs changer year -4 against year -3*/

estimate 'not exp vs unemp -4 vs -3' down_exp2020*co_rel **1** -**1** **0** **0** **0** **0** **0** **0** **0**

**0** **0** **0** **0** **0** **0** **0** **0** **0**

**0** **0** **0** **0** **0** **0** **0** **0** **0**

-**1** **1** **0** **0** **0** **0** **0** **0** **0** /exp e; /*not exposed vs unemployed year -4 against year -3*/

*year -3 vs -1;

estimate 'not exp vs stayer -3 vs -1' down_exp2020*co_rel **0** **1** **0** -**1** **0** **0** **0** **0** **0**

**0** -**1** **0** **1** **0** **0** **0** **0** **0**

**0** **0** **0** **0** **0** **0** **0** **0** **0**

**0** **0** **0** **0** **0** **0** **0** **0** **0** /exp e; /*not exposed vs stayers year -3 against year -1*/

estimate 'not exp vs changer -3 vs -1' down_exp2020*co_rel **0** **1** **0** -**1** **0** **0** **0** **0** **0**

**0** **0** **0** **0** **0** **0** **0** **0** **0**

**0** -**1** **0** **1** **0** **0** **0** **0** **0**

**0** **0** **0** **0** **0** **0** **0** **0** **0** /exp e; /*not exposed vs changer year -3 against year -1*/

estimate 'not exp vs unemp -3 vs 1' down_exp2020*co_rel **0** **1** **0** -**1** **0** **0** **0** **0** **0**

**0** **0** **0** **0** **0** **0** **0** **0** **0**

**0** **0** **0** **0** **0** **0** **0** **0** **0**

**0** -**1** **0** **1** **0** **0** **0** **0** **0** /exp e; /*not exposed vs unemployed year -1 against year 1*/

*year -1 vs 1;

estimate 'not exp vs stayer -1 vs 1' down_exp2020*co_rel **0** **0** **0** **1** **0** -**1** **0** **0** **0**

**0** **0** **0** -**1** **0** **1** **0** **0** **0**

**0** **0** **0** **0** **0** **0** **0** **0** **0**

**0** **0** **0** **0** **0** **0** **0** **0** **0** /exp e; /*not exposed vs stayers year -1 against year 1*/

estimate 'not exp vs changer -1 vs 1' down_exp2020*co_rel **0** **0** **0** **1** **0** -**1** **0** **0** **0**

**0** **0** **0** **0** **0** **0** **0** **0** **0**

**0** **0** **0** -**1** **0** **1** **0** **0** **0**

**0** **0** **0** **0** **0** **0** **0** **0** **0** /exp e; /*not exposed vs changer year -1 against year 1*/

estimate 'not exp vs unemp -1 vs 1' down_exp2020*co_rel **0** **0** **0** **1** **0** -**1** **0** **0** **0**

**0** **0** **0** **0** **0** **0** **0** **0** **0**

**0** **0** **0** **0** **0** **0** **0** **0** **0**

**0** **0** **0** -**1** **0** **1** **0** **0** **0** /exp e; /*not exposed vs unemployed year -1 against year 1*/

*year +1 vs 4;

estimate 'not exp vs stayer +1 vs +4' down_exp2020*co_rel **0** **0** **0** **0** **0** **1** **0** **0** -**1**

**0** **0** **0** **0** **0** -**1** **0** **0** **1**

**0** **0** **0** **0** **0** **0** **0** **0** **0**

**0** **0** **0** **0** **0** **0** **0** **0** **0** /exp e; /*not exposed vs stayers year +1 against year 4*/

estimate 'not exp vs changer +1 vs +4' down_exp2020*co_rel **0** **0** **0** **0** **0** **1** **0** **0** -**1**

**0** **0** **0** **0** **0** **0** **0** **0** **0**

**0** **0** **0** **0** **0** -**1** **0** **0** **1**

**0** **0** **0** **0** **0** **0** **0** **0** **0** /exp e; /*not exposed vs changer year +1 against year 4*/

estimate 'not exp vs unemp +1 vs +4' down_exp2020*co_rel **0** **0** **0** **0** **0** **1** **0** **0** -**1**

**0** **0** **0** **0** **0** **0** **0** **0** **0**

**0** **0** **0** **0** **0** **0** **0** **0** **0**

**0** **0** **0** **0** **0** -**1** **0** **0** **1** /exp e; /*not exposed vs unemployed year +1 against year 4*/

*year -1 vs 4;

estimate 'not exp vs stayer -1 vs +4' down_exp2020*co_rel **0** **0** **0** **1** **0** **0** **0** **0** -**1**

**0** **0** **0** -**1** **0** **0** **0** **0** **1**

**0** **0** **0** **0** **0** **0** **0** **0** **0**

**0** **0** **0** **0** **0** **0** **0** **0** **0** /exp e; /*not exposed vs stayers year -1 against year 4*/

estimate 'not exp vs changer -1 vs +4' down_exp2020*co_rel **0** **0** **0** **1** **0** **0** **0** **0** -**1**

**0** **0** **0** **0** **0** **0** **0** **0** **0**

**0** **0** **0** -**1** **0** **0** **0** **0** **1**

**0** **0** **0** **0** **0** **0** **0** **0** **0** /exp e; /*not exposed vs changer year -1 against year 4*/

estimate 'not exp vs unemp -1 vs +4' down_exp2020*co_rel **0** **0** **0** **1** **0** **0** **0** **0** -**1**

**0** **0** **0** **0** **0** **0** **0** **0** **0**

**0** **0** **0** **0** **0** **0** **0** **0** **0**

**0** **0** **0** -**1** **0** **0** **0** **0** **1** /exp e; /*not exposed vs unemployed year -1 against year 4*/

repeated subject=Lopnr / within=co_rel type=ar;

ods output lsmeans=tempout;

**run**;

*same procedure for count data but changing the distribution from binary to poisson

and outcomes to into N05b_sum, and N05c_sun, respectively;

*including only those that have a purchase on n05b or n05c, respectively, in the analysis,

by employment status after downsizing or unexposed, analysis on N05b and N05c were performed separately;

*example;

*crude;

**proc** **genmod** data= ;

class Lopnr co_rel year;

model N05b_sum = co_rel year / dist=poisson link=log; ** poisson regression **;

lsmeans co_rel / diff cl; ** rate=exp(estimate) **;

estimate '-3 vs -4' co_rel -**1** **1** **0** **0** **0** **0** **0** **0** **0** / exp;

estimate '-1 vs -3' co_rel **0** -**1** **0** **1** **0** **0** **0** **0** **0** / exp;

estimate '1 vs -1' co_rel **0** **0** **0** -**1** **0** **1** **0** **0** **0** / exp;

estimate '4 vs 1' co_rel **0** **0** **0** **0** **0** -**1** **0** **0** **1** / exp;

repeated subject=Lopnr / within=co_rel type=ar;

ods output lsmeans=tempout;

**run**;

*adj;

**proc** **genmod** data= ;

class Lopnr co_rel year kon ageprc edupr pr_sick_hist fampr regionpr ;

model N05b_sum = co_rel year kon ageprc edupr pr_sick_hist fampr regionpr / dist=poisson link=log; ** poisson regression **;

lsmeans co_rel / diff cl; ** rate=exp(estimate) **;

estimate '-3 vs -4' co_rel -**1** **1** **0** **0** **0** **0** **0** **0** **0** / exp;

estimate '-1 vs -3' co_rel **0** -**1** **0** **1** **0** **0** **0** **0** **0** / exp;

estimate '1 vs -1' co_rel **0** **0** **0** -**1** **0** **1** **0** **0** **0** / exp;

estimate '4 vs 1' co_rel **0** **0** **0** **0** **0** -**1** **0** **0** **1** / exp;

repeated subject=Lopnr / within=co_rel type=ar;

ods output lsmeans=tempout;

**run**;

*contrast---

everyone who has made a purchase in the data set to contrast the employment groups against each other;

**proc** **genmod** data= n05b_if;

class Lopnr down_exp2020 co_rel year kon pr_sick_hist ageprc edupr fampr regionpr;

model N05b_sum = down_exp2020 co_rel year kon pr_sick_hist ageprc edupr fampr regionpr down_exp2020*co_rel / dist=poisson link=log;

*year -4 vs -3;

estimate 'not exp vs stayer -4 vs -3' down_exp2020*co_rel **1** -**1** **0** **0** **0** **0** **0** **0** **0**

-**1** **1** **0** **0** **0** **0** **0** **0** **0**

**0** **0** **0** **0** **0** **0** **0** **0** **0**

**0** **0** **0** **0** **0** **0** **0** **0** **0** /exp ; /*not exposed vs stayers year -4 against year -3*/

estimate 'not exp vs changer -4 vs -3' down_exp2020*co_rel **1** -**1** **0** **0** **0** **0** **0** **0** **0**

**0** **0** **0** **0** **0** **0** **0** **0** **0**

-**1** **1** **0** **0** **0** **0** **0** **0** **0**

**0** **0** **0** **0** **0** **0** **0** **0** **0** /exp ; /*not exposed vs changer year -4 against year -3*/

estimate 'not exp vs unemp -4 vs -3' down_exp2020*co_rel **1** -**1** **0** **0** **0** **0** **0** **0** **0**

**0** **0** **0** **0** **0** **0** **0** **0** **0**

**0** **0** **0** **0** **0** **0** **0** **0** **0**

-**1** **1** **0** **0** **0** **0** **0** **0** **0** /exp ; /*not exposed vs unemployed year -4 against year -3*/

*year -3 vs -1;

estimate 'not exp vs stayer -3 vs -1' down_exp2020*co_rel **0** **1** **0** -**1** **0** **0** **0** **0** **0**

**0** -**1** **0** **1** **0** **0** **0** **0** **0**

**0** **0** **0** **0** **0** **0** **0** **0** **0**

**0** **0** **0** **0** **0** **0** **0** **0** **0** /exp ; /*not exposed vs stayers year -3 against year -1*/

estimate 'not exp vs changer -3 vs -1' down_exp2020*co_rel **0** **1** **0** -**1** **0** **0** **0** **0** **0**

**0** **0** **0** **0** **0** **0** **0** **0** **0**

**0** -**1** **0** **1** **0** **0** **0** **0** **0**

**0** **0** **0** **0** **0** **0** **0** **0** **0** /exp ; /*not exposed vs changer year -3 against year -1*/

estimate 'not exp vs unemp -3 vs 1' down_exp2020*co_rel **0** **1** **0** -**1** **0** **0** **0** **0** **0**

**0** **0** **0** **0** **0** **0** **0** **0** **0**

**0** **0** **0** **0** **0** **0** **0** **0** **0**

**0** -**1** **0** **1** **0** **0** **0** **0** **0** /exp ; /*not exposed vs unemployed year -1 against year 1*/

*year -1 vs 1;

estimate 'not exp vs stayer -1 vs 1' down_exp2020*co_rel **0** **0** **0** **1** **0** -**1** **0** **0** **0**

**0** **0** **0** -**1** **0** **1** **0** **0** **0**

**0** **0** **0** **0** **0** **0** **0** **0** **0**

**0** **0** **0** **0** **0** **0** **0** **0** **0** /exp ; /*not exposed vs stayers year -1 against year 1*/

estimate 'not exp vs changer -1 vs 1' down_exp2020*co_rel **0** **0** **0** **1** **0** -**1** **0** **0** **0**

**0** **0** **0** **0** **0** **0** **0** **0** **0**

**0** **0** **0** -**1** **0** **1** **0** **0** **0**

**0** **0** **0** **0** **0** **0** **0** **0** **0** /exp ; /*not exposed vs changer year -1 against year 1*/

estimate 'not exp vs unemp -1 vs 1' down_exp2020*co_rel **0** **0** **0** **1** **0** -**1** **0** **0** **0**

**0** **0** **0** **0** **0** **0** **0** **0** **0**

**0** **0** **0** **0** **0** **0** **0** **0** **0**

**0** **0** **0** -**1** **0** **1** **0** **0** **0** /exp ; /*not exposed vs unemployed year -1 against year 1*/

*year 1 vs 4;

estimate 'not exp vs stayer 1 vs 4' down_exp2020*co_rel **0** **0** **0** **0** **0** **1** **0** **0** -**1**

**0** **0** **0** **0** **0** -**1** **0** **0** **1**

**0** **0** **0** **0** **0** **0** **0** **0** **0**

**0** **0** **0** **0** **0** **0** **0** **0** **0** /exp ; /*not exposed vs stayers year 1 against year 4*/

estimate 'not exp vs changer 1 vs 4' down_exp2020*co_rel **0** **0** **0** **0** **0** **1** **0** **0** -**1**

**0** **0** **0** **0** **0** **0** **0** **0** **0**

**0** **0** **0** **0** **0** -**1** **0** **0** **1**

**0** **0** **0** **0** **0** **0** **0** **0** **0** /exp ; /*not exposed vs changer year 1 against year 4*/

estimate 'not exp vs unemp 1 vs 4' down_exp2020*co_rel **0** **0** **0** **0** **0** **1** **0** **0** -**1**

**0** **0** **0** **0** **0** **0** **0** **0** **0**

**0** **0** **0** **0** **0** **0** **0** **0** **0**

**0** **0** **0** **0** **0** -**1** **0** **0** **1** /exp ; /*not exposed vs unemployed year 1 against year 4*/

*year -1 vs 4;

estimate 'not exp vs stayer -1 vs 4' down_exp2020*co_rel **0** **0** **0** **1** **0** **0** **0** **0** -**1**

**0** **0** **0** -**1** **0** **0** **0** **0** **1**

**0** **0** **0** **0** **0** **0** **0** **0** **0**

**0** **0** **0** **0** **0** **0** **0** **0** **0** /exp ; /*not exposed vs stayers year -1 against year 4*/

estimate 'not exp vs changer -1 vs 4' down_exp2020*co_rel **0** **0** **0** **1** **0** **0** **0** **0** -**1**

**0** **0** **0** **0** **0** **0** **0** **0** **0**

**0** **0** **0** -**1** **0** **0** **0** **0** **1**

**0** **0** **0** **0** **0** **0** **0** **0** **0** /exp ; /*not exposed vs changer year -1 against year 4*/

estimate 'not exp vs unemp -1 vs 4' down_exp2020*co_rel **0** **0** **0** **1** **0** **0** **0** **0** -**1**

**0** **0** **0** **0** **0** **0** **0** **0** **0**

**0** **0** **0** **0** **0** **0** **0** **0** **0**

**0** **0** **0** -**1** **0** **0** **0** **0** **1** /exp ; /*not exposed vs unemployed year -1 against year 4*/

repeated subject=Lopnr / within=co_rel type=ar;

ods output lsmeans=tempout;

**run**;
