# Supplementary material for: Downsizing and purchases of psychotropic drugs: A longitudinal study of stayers, changers and unemployed
Source: PLoS One. 2023 Dec 8;18(12):e0295383. doi: 10.1371/journal.pone.0295383 (PMC10707493; doi:10.1371/journal.pone.0295383)
Supplement: S1 Table — Statistically significant differences between exposed and not exposed are expressed with * in mutually adjusted models. (DOCX) [file pone.0295383.s014.docx]

| **Table. 2 Rate Ratios (RR) for purchases of prescription anxiolytics and sedatives and their 95% Confidence Interval (CI) in relation to a downsizing event, comparing changes over time within groups in analyses stratified by downsizing and employment status after downsizing. Any statistically significant differences between exposed and not exposed are expressed with * in mutually adjusted models.** | | | | | | | | |
| --- | --- | --- | --- | --- | --- | --- | --- | --- |
|  | Early pre-downsizing  ‘-3 to -4’^d^ | | Late pre-downsizing ‘-1 to -3’^e^ | | Peri-downsizing  ’+1 to -1’^f^ | | Post-downsizing  ’+4 to +1’^g^ | |
|  | RR | 95% CI | RR | 95% CI | RR | 95% CI | RR | 95% CI |
| **Anxiolytics** |  |  |  |  |  |  |  |  |
| Adjusted |  |  |  |  |  |  |  |  |
| *Not exposed* | 1,00 | 0,98- 1,02 | 1,02 | 0,99- 1,04 | 1,03 | 1,00- 1,05 | 1,02 | 0,98-1,05 |
| *Unemployed* | 0,99 | 0,92-1,07 | 1,16* | 1,06- 1,27 | 0,95* | 0,87- 1,04 | 1,04 | 0,93- 1,17 |
| *Stayer* | 1,01 | 0,97-1,05 | 1,07 | 1,02- 1,13 | 1,08 | 1,04- 1,13 | 1,09* | 1,03- 1,15 |
| *Changer* | 1,03 | 0,98- 1,08 | 1,08 | 1,01- 1,15 | 1,11 | 1,05-1,18 | 1,13 | 1,05- 1,22 |
| **Sedatives** |  |  |  |  |  |  |  |  |
| Adjusted^c^ |  |  |  |  |  |  |  |  |
| *Not exposed* | 1,00 | 0,99-1,02 | 1,02 | 1,00-1,04 | 1,02 | 1,00-1,04 | 1,04 | 1,021,07 |
| *Unemployed* | 1,03 | 0,98-1,09 | 1,15* | 1,071,23 | 0,99* | 0,921,07 | 1,06* | 0,961,16 |
| *Stayer* | 0,98 | 0,96- 1,01 | 1,06 | 1,02-1,10 | 1,06* | 1,02-1,09 | 1,08 | 1,03-1,13 |
| *Changer* | 1,00 | 0,95-1,04 | 1,05 | 1,00-1,10 | 1,06 | 1,02-1,11 | 1,13* | 1,07-1,20 |
