# Supplementary material for: Downsizing and purchases of psychotropic drugs: A longitudinal study of stayers, changers and unemployed
Source: PLoS One. 2023 Dec 8;18(12):e0295383. doi: 10.1371/journal.pone.0295383 (PMC10707493; doi:10.1371/journal.pone.0295383)
Supplement: Supplementary file 3 [file pone.0295383.s002.docx]

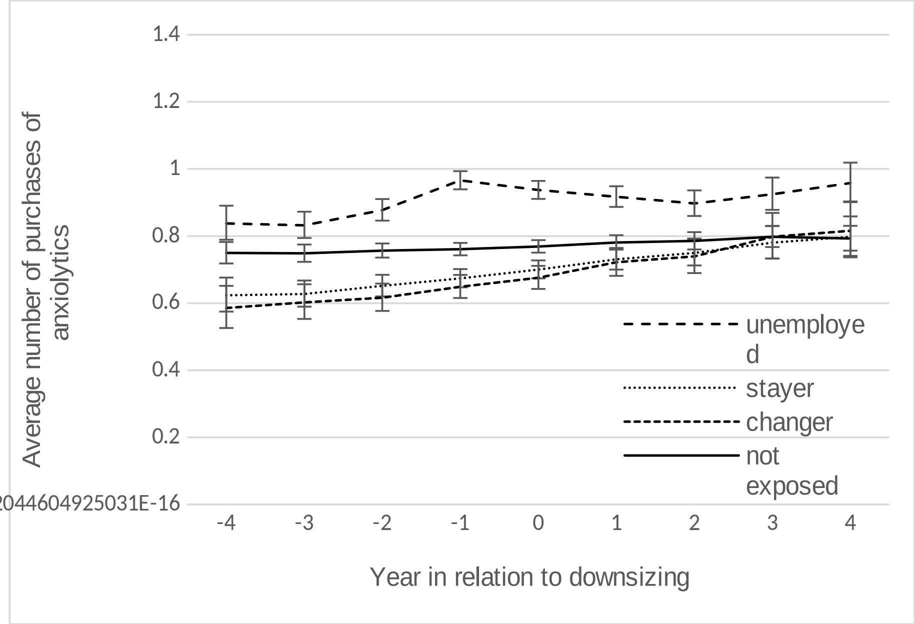

1.4
1.2
1
0.8
0.6
0.4
0.2
-2.22044604925031E-16
-4
-3
-2
-1
0
1
2
3
4
Average number of purchases of anxiolytics
Year in relation to downsizing
unemployed
stayer
changer
not exposed
