# Supplementary material for: Downsizing and purchases of psychotropic drugs: A longitudinal study of stayers, changers and unemployed
Source: PLoS One. 2023 Dec 8;18(12):e0295383. doi: 10.1371/journal.pone.0295383 (PMC10707493; doi:10.1371/journal.pone.0295383)
Supplement: Supplementary file 9 [file pone.0295383.s008.docx]

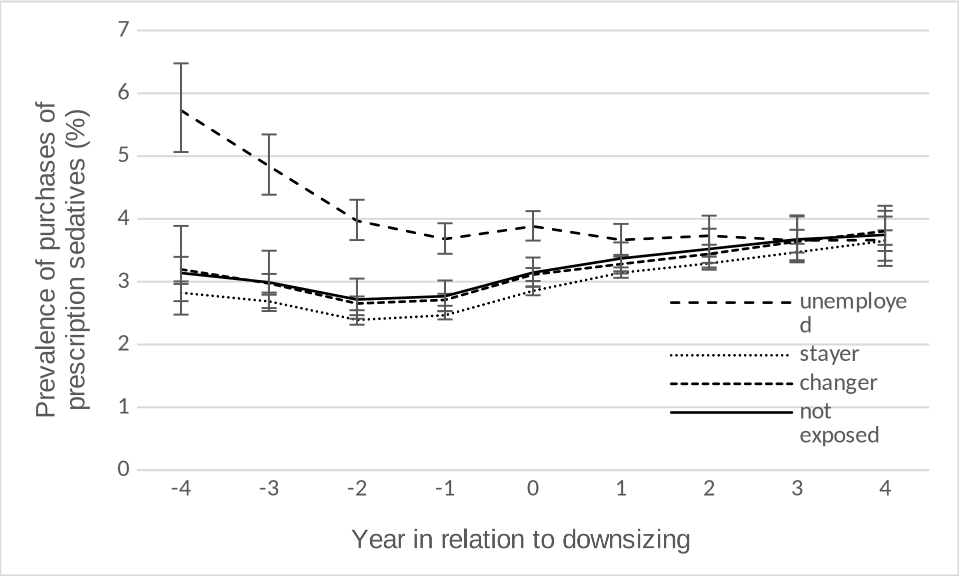

7
6
5
4
3
2
1
0
-4
-3
-2
-1
0
1
2
3
4
Prevalence of purchases of prescription sedatives (%)
Year in relation to downsizing
unemployed
stayer
changer
not exposed
