# Supplementary material for: Downsizing and purchases of psychotropic drugs: A longitudinal study of stayers, changers and unemployed
Source: PLoS One. 2023 Dec 8;18(12):e0295383. doi: 10.1371/journal.pone.0295383 (PMC10707493; doi:10.1371/journal.pone.0295383)
Supplement: Attachment — Submitted filename: Rebuttal letter PLOS One.docx [file pone.0295383.s016.docx]

Journal Requirements:

1)  Citation of the retracted paper (Introduction and Reference list): The text addressing the retraction in the Introduction text is suitable, but the citation of the retraction and original publication is not correct. The in-text citations in the Introduction should refer to both the retracted/original article and the retraction notice, which should be listed as follows in the References list:

17. Blomqvist S, Alexanderson K, Vahtera J, Westerlund H, Magnusson Hanson LL (2018) Downsizing and purchases of psychotropic drugs: A longitudinal study of stayers, changers and unemployed. PLoS ONE 13(8): e0203433. <https://doi.org/10.1371/journal.pone.0203433> (**Retracted**)

18. Blomqvist S, Alexanderson K, Vahtera J, Westerlund H, Magnusson Hanson LL (2023) Retraction: Downsizing and purchases of psychotropic drugs: A longitudinal study of stayers, changers and unemployed. PLOS ONE 18(4): e0285004. <https://doi.org/10.1371/journal.pone.0285004>

When correcting this, please also update the citations and referencing for the articles currently listed as reference numbers 18+.

Thank you for the clarification of how to correctly cite the different versions. We have now updated the manuscript according to the requirements and further updated the subsequent refences in the list after adding reference no. 18, see. p. 4 in the introduction and the reference list on p. 27, (manuscript version w track changes)

2) Analysis scripts: please could the scripts used for the analyses in the study by provided either in a Supplementary Information file or via a public repository.

We have now included the analysis scripts as Supplementary Information, see file:

**S1 Appendix. Analysis script for GEE methods**

And the following note included in the method section on p. 9:

“See S1 Appendix for the SAS 9.4 scripts used for these analyses.”


[Note: HTML markup is below. Please do not edit.]

Reviewers' comments:

Reviewer's Responses to Questions

**Comments to the Author**

1. If the authors have adequately addressed your comments raised in a previous round of review and you feel that this manuscript is now acceptable for publication, you may indicate that here to bypass the “Comments to the Author” section, enter your conflict of interest statement in the “Confidential to Editor” section, and submit your "Accept" recommendation.

Reviewer #2: All comments have been addressed

Reviewer #3: All comments have been addressed

2. Is the manuscript technically sound, and do the data support the conclusions?

The manuscript must describe a technically sound piece of scientific research with data that supports the conclusions. Experiments must have been conducted rigorously, with appropriate controls, replication, and sample sizes. The conclusions must be drawn appropriately based on the data presented.

Reviewer #2: Yes

Reviewer #3: Yes

3. Has the statistical analysis been performed appropriately and rigorously?

Reviewer #2: Yes

Reviewer #3: Yes

4. Have the authors made all data underlying the findings in their manuscript fully available?

The [PLOS Data policy](http://www.plosone.org/static/policies.action#sharing) requires authors to make all data underlying the findings described in their manuscript fully available without restriction, with rare exception (please refer to the Data Availability Statement in the manuscript PDF file). The data should be provided as part of the manuscript or its supporting information, or deposited to a public repository. For example, in addition to summary statistics, the data points behind means, medians and variance measures should be available. If there are restrictions on publicly sharing data—e.g. participant privacy or use of data from a third party—those must be specified.

Reviewer #2: No

Reviewer #3: No

5. Is the manuscript presented in an intelligible fashion and written in standard English?

PLOS ONE does not copyedit accepted manuscripts, so the language in submitted articles must be clear, correct, and unambiguous. Any typographical or grammatical errors should be corrected at revision, so please note any specific errors here.

Reviewer #2: Yes

Reviewer #3: Yes

6. Review Comments to the Author

Please use the space provided to explain your answers to the questions above. You may also include additional comments for the author, including concerns about dual publication, research ethics, or publication ethics. (Please upload your review as an attachment if it exceeds 20,000 characters)

Reviewer #2: Dear Authors,
Thank you for your response and for considering some of my comments. I understand that you could not consider all comments, because this paper is a special case (with its retraction history) and that you had been instructed by Plos One how to handle this. I do not have further comments or requests.

Thank you for taking the time to read and review our manuscript.

Reviewer #3: Thank you for this opportunity to revisit your article concerning mental health and workplace downsizing.

I agree with what both the authors and the reviewers stated in R1: This is a difficult paper to review as it has already undergone the process once. Also, I tend to agree with the authors that as the original paper with the error in it has already been cited, it would be best to refrain from making large changes to the paper. To more complicate things, I did not participate in the first round of peer reviews and most of the points I would have raised and were already made by the two reviewers participating in the first round and then adequately addressed by the authors. I will attempt to review the paper as requested, but with the previous round in mind.

I think the authors have done a good job of describing the error in the original publication. I agree with what prior reviewers already suggested concerning the title, as it would be clearer to the reader if the retracted paper and the current version had separate titles. But this appears to be concern for the PLOS one team and not the authors.

Thank you and we would also like to refer this issue about the title to the PLOS one team and how titles for retraction and resubmissions are normally dealt with.

Several other points were also made in the previous round of reviews which I believe the authors have now adequately addressed, eg. that concerning the inclusion of antidepressants and the cut offs for workplace downsizing. I would have suggested to present results for both anxiolytics/sedatives and antidepressants from one set of data and analyses (addressed both together and separately), as well as running the models for both anxiolytics and sedatives together. However, the authors make a good case for their choice to to adhere to the original analyses.

Yes, we acknowledge that improvement of the paper can be done and these are all good suggestions. However, as noted already, for this particular case we would like to stay as close to the original version as possible and not introduce new types of analysis since the old version has already been cited.

Minor concerns

Table 3. The title is: "Odds Ratios (OR) for purchases of prescription anxiolytics and sedatives and their 95% Confidence Interval (CI) in relation to the downsizing event, comparing changes over time within groups in analyses stratified by downsizing and employment status after downsizing. Any statistically significant differences between exposed and not exposed are expressed with * in mutually adjusted models. All analysis are
stratified by previous sickness absence." Would it be clearer if this was: "Odds Ratios (OR) for purchases of prescription anxiolytics and sedatives and their 95% Confidence Interval (CI) in relation to the downsizing event, comparing changes over time within groups in analyses stratified by downsizing and employment status after downsizing, and previous sickness absence. Any statistically significant differences between exposed and not exposed are expressed with * in mutually adjusted models."

Yes, thank you, this reads a lot smoother. We have now updated the title according to your suggestion. See page 18, manuscript version w track changes.

Discussion. The paper would have benefited from a brief discussion on the public health or clinical relevance of the results. What are the implications of the somewhat modest associations found here? But as you have already made multiple changes following reviewers suggestions and as the space is limited, I am happy with the discussion as is.

In conclusions you write: "To conclude, this study supports an association between downsizing and purchases of
prescribed psychotropic drugs. The association was observed before the event but especially
prominent --" I'm not a native speaker, but I struggled with this section. Would something to the following effect be clearer to the reader: "This study supports an association between downsizing and purchases of
prescribed psychotropic drugs. The association was especially prominent for the stayers before the event, and they also increased their purchases during and after downsizing. --- further implies that organizational downsizing can have long term mental health consequences for those who stay in the organization, indicating a need for health promoting measures."

We see your point but we also believe that the suggestion points in a slightly different direction than what we have intended. The text alteration seems to suggest that stayers were particularly affected prior to the event but this was not the case for this group, they were rather affected during and after the event- although some effect seemed to exist prior to the event too (mostly for those with a previous sickness absence). However, our original formulation was not optimal either. We therefore suggest that it is worded in following way instead (on page 24-25 in the manuscript w tracked changes):

“To conclude, this study supports an association between downsizing and purchases of prescribed psychotropic drugs. The results indicating an increase in psychotropic drug purchases prior to the downsizing event among changes and those who became unemployed after the downsizing implies that the anticipation-phase is a stressful time in the downsizing process for the exposed employees. The results however, suggested an even more prominent increase psychotropic drug purchases across the period of downsizing and the following years among those who continued to work in the same workplace, i.e., the stayers. This also implies that an organizational downsizing can have long-term consequences on mental health for those who stay in the organization, which did not appear to be attributed to health selection, indicating a need for preventive measures.”

Altogether in the discussion, conclusions and abstract I sometimes found it difficult to say what your main conclusions for the leavers and the unemployed were. Perhaps these two groups could be included the conclusions, as well as add more detail on the main findings in the beginning of the discussion or the abstract?

See our suggestion above, where we’ve tried to address this issue by more explicitly include how unemployed and changers were affected in the overall conclusion. We also revised the abstract slightly to be more explicit about the changers and the unemployed, i.e., the leavers. See the abstract and below:

“This study indicates that being exposed to downsizing is associated with increased use of sedatives and anxiolytics, before the event among those who leave, but especially thereafter for employees who stay in the organization.”

We further added the following sentence in the discussion section concerning the most important findings for these two groups, which is placed as the second paragraph in the discussion section, p. 20

“During the period prior to the downsizing event, often referred to as the anticipation phase, it was primarily the unemployed and the changer groups that seemed to experience an increase in psychotropic drug purchases. The findings are in accordance with previous studies showing that the anticipation phase before a job change or unemployment has predominantly detrimental health effects [29-31].”

7. PLOS authors have the option to publish the peer review history of their article ([what does this mean?](https://journals.plos.org/plosone/s/editorial-and-peer-review-process#loc-peer-review-history)). If published, this will include your full peer review and any attached files.


If you choose “no”, your identity will remain anonymous but your review may still be made public.


**Do you want your identity to be public for this peer review?** For information about this choice, including consent withdrawal, please see our [Privacy Policy](https://www.plos.org/privacy-policy).

Reviewer #2: No

Reviewer #3: No

[NOTE: If reviewer comments were submitted as an attachment file, they will be attached to this email and accessible via the submission site. Please log into your account, locate the manuscript record, and check for the action link "View Attachments". If this link does not appear, there are no attachment files.]

While revising your submission, please upload your figure files to the Preflight Analysis and Conversion Engine (PACE) digital diagnostic tool, <https://pacev2.apexcovantage.com/>. PACE helps ensure that figures meet PLOS requirements. To use PACE, you must first register as a user. Registration is free. Then, login and navigate to the UPLOAD tab, where you will find detailed instructions on how to use the tool. If you encounter any issues or have any questions when using PACE, please email PLOS at [figures@plos.org](mailto:figures@plos.org). Please note that Supporting Information files do not need this step.
